# Nanostructures formed by displacement of porous silicon with copper: from nanoparticles to porous membranes

**DOI:** 10.1186/1556-276X-7-477

**Published:** 2012-08-23

**Authors:** Hanna Bandarenka, Sergey Redko, Aleksandr Smirnov, Andrei Panarin, Sergei Terekhov, Paolo Nenzi, Marco Balucani, Vitaly Bondarenko

**Affiliations:** 1 Department of Micro- and Nanoelectronics, Belarusian State University of Informatics and Radioelectronics, 6 Brovka St., Minsk, 220013, Belarus; 2 B.I. Stepanov Institute of Physics, National Academy of Sciences of Belarus, 68 Nezalezhnasti Ave, Minsk, 0, Belarus; 3 Department of Information Engineering, Electronics and Telecommunications, University “Sapienza”, 18 Eudossiana St., Rome, 00184, Italy

**Keywords:** Porous silicon, Copper, Displacement deposition, Nanostructures

## Abstract

The application of porous silicon as a template for the fabrication of nanosized copper objects is reported. Three different types of nanostructures were formed by displacement deposition of copper on porous silicon from hydrofluoric acid-based solutions of copper sulphate: (1) copper nanoparticles, (2) quasi-continuous copper films, and (3) free porous copper membranes. Managing the parameters of porous silicon (pore sizes, porosity), deposition time, and wettability of the copper sulphate solution has allowed to achieve such variety of the copper structures. Elemental and structural analyses of the obtained structures are presented. Young modulus measurements of the porous copper membrane have been carried out and its modest activity in surface enhanced Raman spectroscopy is declared.

## Background

Despite its long-standing discovery, porous silicon (PS) has been attracting a great attention as a breakthrough material with exceptional characteristics for microelectronics, integrated optoelectronics, microelectromechanical systems (MEMS), layer transfer technology, solar and fuel cells, biomedicine, etc. [1]. Partially, this is because of the opportunity to easily vary the properties of PS in wide ranges by introducing different materials into its pores [2]. Indeed, the use of an array of ordered pores as a template can provide the creation of specific composite structures with novel electrical, optical, magnetic, plasmonic, and other features [3-5]. Among many, an interest on nanocomposites fabricated by immersion of PS into aqueous solution of copper salt has not been attenuating for more than a decade [6-8], though the mechanism of Cu immersion deposition on bulk monocrystalline silicon had been studied much earlier because wet chemical cleaning in H_2_O- and hydrofluoric acid (HF)-based solutions containing an extremely low concentration of copper ions has resulted in the adhesion of copper contaminants on Si wafers [9]. To prevent the presence of undesirable Cu traces on Si, the mechanism of copper adhesion has been studied and understood [10,11]. Because of their positive redox potential, copper ions have been found to attract electrons from silicon, resulting in simultaneous copper reduction and Si oxidation [10]. In that way, the nucleation and growth of Cu precipitates with diameters of few nanometers occur [11-13]. Later, a number of studies have been carried out to fabricate copper films by immersion of bulk silicon in a solution with higher concentration of copper ions [14,15]. It promotes growth of copper precipitates to islands which then increase in sizes and coalesce together forming a quasi-continuous film [15,16]. Such films have been representing as suitable candidates for IC interconnections and MEMS technology due to their low resistivity, their selectivity of deposition between silicon and dielectric mask, as well as the simplicity and cost-effectiveness of the fabrication process, which does not require high temperature, special complex equipment, and illumination [14,15]. However, to obtain reliable adhesion of immersion Cu films to Si, it is necessary to use 350°C in annealing [14]. To solve the problem in an easier way, the authors of the papers [17,18] have proposed to form a thin layer of PS before copper deposition. Deep penetration of copper atoms into porous layer during immersion deposition results in the formation of a Cu/PS composite, providing several times of increasing copper film adhesion. This is another advantage of the immersion method because during evaporation or sputtering, depositing copper atoms are located at the entrances of pores [2].

On the other hand, PS is traditionally used as a direct-bandgap semiconductor (in contrast to bulk indirect-bandgap Si) that allows integration of optoelectronic devices with Si technology [1]. Actually, PS is known to demonstrate visible, red photoluminescence, but introduction of copper nanoparticles (NPs) in its porous volume promotes obtaining emission in other wavelengths [19]. Prospects of easy variation of the PS luminescence have significantly increased the interest in studying copper immersion deposition in PS. The subsequent research established the influence of the inner composition of PS (SiO_2_, SiH_x_, O_y_SiH_x_) on chemical reactions [6] and the inhibition of deposition in the presence of halogen ions [7]. Other specific features of immersion Cu/PS is an activity in surface enhanced Raman spectroscopy (SERS) [20] which is one of the most sensitive methods in analytical chemistry, biomedicine, ecology, etc. Unfortunately, Cu/PS SERS-active substrates have not been widely studied yet in comparison with other competing porous substrates based on anodic aluminum oxide [21].

All mentioned works on the immersion deposition of Cu on PS have applied simple aqueous solutions of copper salts accompanied by the formation of SiO_2_ under a copper deposit which stops the redox reaction and prevents the dissolution of porous template. At the same time, it has limited the number of nanoscale structures which could be formed by the immersion method. Recently, turning back to the study of copper contaminations on bulk Si, addition of HF to the solution for copper deposition on PS has been proposed [22]. HF allows SiO_2_ removal and continuous deposition of copper, as well as silicon dissolution. This process is usually called displacement deposition because of the nonstop substitution of the substrate's atoms with the metal's atoms [15]. Some previous works have shown that copper deposits by displacement on the surface of PS in the form of crystalline NPs [23]. The pore channels limit the size of NPs, while on the outer PS surface, copper particles can be an order of magnitude greater [24]. In fact, the final material of copper displacement deposition on PS represents the layer of the Cu/PS nanocomposite covered with quasi-continuous copper film. The initial stages of deposition are accompanied by the formation of copper particles of 2-nm diameter inheriting the crystallographic orientation of the PS skeleton [25]. Further copper particles growth leads to the (111) prevalence orientation of the copper deposit [24,26]. In [24], the outer surface of PS of 55% porosity has been used as a template for the growth of copper particles of controllable sizes. However, the authors have been faced with the fact that copper deposits according to the island growth mechanism of thin films (like in the case of bulk Si). As the level of PS surface coverage with copper particles reached the critical value, gradual formation of quasi-continuous Cu film has been observed. To achieve the growth of separated Cu particle arrays on the PS substrate of required dimensions, the authors reduced the solution temperature and used alcohol as the wetting agent. Nevertheless, easy and controllable managing of the morphology and structure of the Cu deposit on PS is still an urgent target as it helps to develop new, effective, and simple technology both for ohmic contact and Schottky-like structures, and conductive films of extremely high adhesion to Si and PS [17,26]. It is notable that to date, positive results of the displacement method might be presented. Combined technology of double-layered PS and copper displacement deposition has been successfully tested for the measurement of PS mechanical strength [17] and manufactured to form compliant contact arrays for probe cards [27]. Remarkably, a spiral of thick, porous copper membrane on a flexible silicone substrate reported to be fabricated by displacement technique from PS has been found to promote drug electroporation [28]. However, fabrication regimes and the morphology of the original PS have not been opened as the authors have referred to the paper [18] which reported the full conversion of PS of only 1-μm thickness into a copper layer, whereas further successful application of such metal porous structures strongly depends on the detailed understanding of its formation mechanism and properties. One of the strong needs is mechanical strength data of the membrane for electroporation because to be in good contact with the surface of treated living tissue, it should have flexible stability.

In the present work, we have proposed to vary parameters of PS to fabricate by displacement technique copper NPs of controllable dimensions as well as thick, porous copper membrane. We have carried out measurements of the Young modulus of the obtained copper membrane. In addition, modest SERS activity of the copper porous membrane has been declared.

## Methods

Czochralski monocrystalline silicon wafers were used as initial substrates to form PS templates of different thicknesses and porosities. The characteristics of Si wafers and parameters of PS are presented in Table 1. The Si wafers were cleaned for 10 min with a hot (75°C) solution of NH_4_OH, H_2_O_2_, and H_2_O mixed in a volume ratio of 1:1:4. Then, the wafers were dried in a centrifuge and cut into a number of 3 × 3-cm rectangular samples. Just before PS formation, each experimental sample was immersed into 5% HF solution for 30 s to remove native silicon oxide. Immediately after oxide removal, the Si sample was placed in an electrolytic cell made of Teflon. The active O-ring opening of the cell had an area of 3 cm^2^. Uniform PS layers were formed by electrochemical anodization of silicon samples in a solution of HF (45%), H_2_O, and C_3_H_7_OH (or DMSO) mixed in a 1:3:1 volume ratio. A spectrally pure graphite disk was used as a contact electrode to the back side of the samples during the electrochemical treatment. Platinum spiral wire was used as a cathode electrode. Anodization was performed at a current density of 7 to 80 mA/cm^2^ for different time periods. Detailed description of the structure and morphology of PS1 to PS3 might be found in [18,29]. They represent arrays of ordered pore channels which are oriented perpendicular to the surface of the Si substrate. The diameter of pores usually varies from 20 to 50 nm. According to international classification [30], it is a material of mesoporous media. The PS4 type has not been previously observed in the literature, so it is firstly described below in the present paper. Characteristics of initial Si wafers and parameters of formed PS are presented in Table 1.

**Table 1:** Si wafer characteristics and PS parameters

**Template number**	**Si wafer doping**	**Si wafer resistivity, *ρ***	**Porosity of PS, *p***	**Thickness of PS, *d***
		**(Ω·cm)**	**(%)**	**(μm)**
PS1	Antimony	0.01	50 to 55	1
PS2			80 to 85	
PS3			80 to 85	7
PS4	Boron	0.3	60 to 65	2.5 to 3

After PS formation, the HF solution was removed, and the electrolytic cell was thoroughly rinsed with deionized water for 3 min and then with C_3_H_7_OH for 5 min. The cell was filled with the solution for copper deposition for 4 to 7200 s at 25°C. We used two solutions for the copper displacement deposition: (1) basic CuSO_4_·5H_2_O + 0.005 M HF (45%) aqueous solution and (2) 0.025 M CuSO_4_·5H_2_O + 0.005 M HF (45%) + 0.1 M C_3_H_7_OH aqueous solution of improved wettability. To stop the deposition process, the solution was poured from the cell. Finally, the sample with a Cu/PS layer was rinsed three times for 30 s with deionized water, dried in air for 30 min, and removed from the cell.

The morphology and structure of the samples were studied with a scanning electron microscope (SEM; Hitachi S-4800, Chiyoda-ku, Japan) with a resolution of 1 nm. The elemental composition of the samples was determined using a Cambridge Instruments Stereoscan-360 SEM (Cambridge, UK) with a Link Analytical AN 10000 energy-dispersive X-ray analyzer (Redwood, CA, USA). The diameter of the focused electron beam was no more than 1 μm, the atomic mass accuracy did not extend 0.1%, and the depth of the analysis was 1.3 to 1.5 μm under 20 keV. The equipment used to conduct electrochemical processes was the AUTOLAB PGSTAT302n potentiostat/galvanostat (Utrecht, The Netherlands). Gravimetric method was applied to determine the porosity of PS and copper membrane. Mass measurements were erformed with a Sartorius CP225D micro/analytical electronic balance (Goettingen, Germany). The instrumental mass error was about 10 μg. The phase composition of the samples was determined by X-ray diffraction (XRD) using CuKα radiation (X-ray wavelength *λ* = 0.15406 nm).

**Figure 1:**
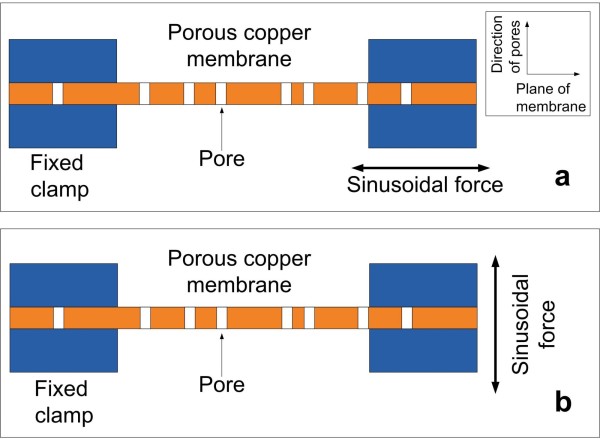
**Schematic view of configurations for Young modulus measurements.** (**a**) Tension configuration *E*_*⊥*_, (**b**) single cantilever configuration *E*_*∥*_.

Young modulus measurements of the porous copper membrane were performed in air by means of a PerkinElmer DMA8000 system (Waltham, MA, USA) in the temperature range of −100°C to 100°C. In all experiments, the sample was forced by an external sinusoidal stress at a frequency of 1 Hz. Measurements were conducted with the sample mounted in two different geometries in order to obtain the elastic modulus either perpendicular to the pore direction, *E*_*∥*_, that is along the plane of the membrane, or along the pore direction, *E*_*⊥*_, that is perpendicular to the plane of the membrane. In the first case, the so called ‘tension’ configuration was used as it is shown in Figure 1: the sample was clamped between a fixed end and a mobile part, where the oscillating force extending the membrane in its plane was applied. A static load was superimposed to the oscillating force in order to avoid buckling. In the second configuration, the so called ‘single cantilever’ experiment was performed: the sample was clamped between a fixed part and a mobile clamp which applies the force along the direction parallel to the pores and perpendicular to the plane of the membrane. The relative strains were kept below 1.2% and 0.4% in the tension and in the cantilever configuration, respectively. Some preliminary measurements were performed to be sure that the sample was in the linear region of the stress–strain curve.

SERS activity of the porous copper membrane was tested using water-soluble cationic Cu(II)-tetrakis(4-*N*-methylpyridyl)porphyrin (CuTMpyP4) as an analyte compound. For the SERS measurements, a 0.02-ml drop of the 10^−6^ М porphyrin solution was poured on the porous copper membrane. After drying in air, a round spot of 1-cm diameter was observed on the copper surface. Raman spectra were registered with the spectrometers SpectraPro 500 I and T64000 (Jobin-Yvon, Milan, Italy), equipped with CCD detectors. The sources of continuous excitation were a Liconix helium-cadmium laser (*λ* = 441.6 nm; Santa Clara, CA, USA) and a semiconductor laser (*λ* = 532 nm). The accuracy of the frequencies in the spectra did not exceed 1 cm^−1^. SERS spectra were recorded upon continuous rotation of the sample for signal averaging and prevention of porphyrin destruction.

## Results and discussions

The deposition process was visually accompanied by gradual color change of the surface of PS from black to red which is typical for copper. Gas bubbles released from the surface of the sample were also observed. The activity of the gas evolution was weakened with the increase of deposition time. According to [11], the released gas is hydrogen which is a product of the redox reactions. The decrease of its evolution means a slowing of the process.

**Figure 2:**
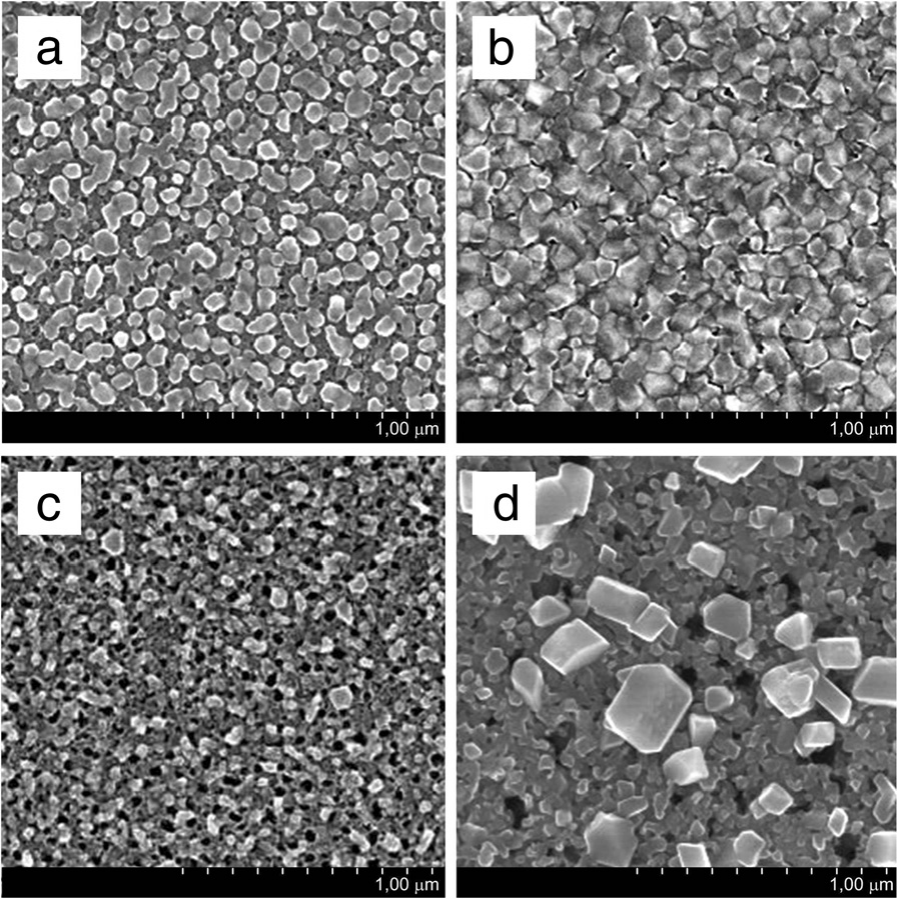
**SEM top views of Cu NPs.** Cu NPs were grown on PS with a thickness of 1 μm and porosity of 50% to 55% (**a**, **b**) and 80% to 85% (**c**, **d**) by displacement deposition for 4 s (**a**, **c**) and 180 s (**b**, **d**).

**Figure 3:**
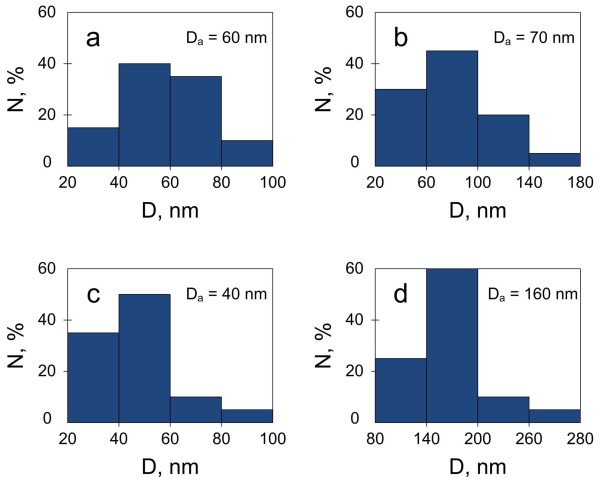
**Size distribution histograms of copper NPs.** Histograms were calculated for Cu NPs grown on PS with a thickness of 1 μm and porosity of 50% to 55% (**a**, **b**) and 80% to 85% (**c**, **d**) by displacement deposition for 4 s (**a**, **c**) and 180 s (**b**, **d**).

Figure 2 shows top-view SEM images of PS1 (a, b) and PS2 (c, d), both of which were immersed into the basic solution of copper sulphate for 4 s (a, c) and 180 s (b, d). The maximum deposition time (180 s) was chosen because at that moment, hydrogen release almost stopped for both types of PS, i.e., the redox process was too weak for the valuable amount of copper deposition or finished. It is seen that the porous surface is covered with copper particles of various dimensions. The phase composition of copper deposited by displacement method on PS1 and PS2 was assessed in earlier research from their XRD patterns [31]. It was found that all copper deposits had a polycrystalline nature with prevalent growth of (111)-oriented crystals. Here, we analyzed the XRD patterns (presented in [24]) and, following Scherrer's formula, calculated that Cu particles consist of 2-nm nanocrystals. That is in good agreement with the known data of EBSD analysis [25]. Figure 3 presents particle size distribution histograms counted from the SEM images (Figure 2). The accuracy of size evaluation did not exceed 2 nm. Early stages of deposition for both types of PS resulted in the growth of separated copper particles (Figure 2a,c) with diameters which varied in the range of 20 to 100 nm (Figure 3a,c). However, higher porosity of PS template (PS2) inhibits the process of particle size increase (Figure 2c) as the average diameter of Cu particles on PS2 is slightly less than that on PS1 (Figure 3a,c). It is probably caused by less number of electrons from the smaller Si elements of the PS2 skeleton (see the structure of PS in [31]). Further deposition of Cu on PS1 led to a slight increase in the diameter of copper particles (Figure 3b), and their coalescence (Figure 2b) resulted in a tightly packed film formation. On the other hand, Cu deposition for 180 s significantly shifted the particle size range from 20 to 100 nm to 80 to 280 nm (Figure 3c,d), while the morphology of copper deposit still represents the separated particles. At the same time, the view of the underlying porous material differs in comparison with Figure 2c: the sponge converted into a grainy porous structure. To understand the changes, we recognized the paper [18] that reported AES analysis of Cu/PS1 and Cu/PS2 formed for 180 s of Cu deposition. The first one represented a nanocomposite with the amount of Cu decreasing from 95% to 15% at pore deepening, while the second structure contained almost no Si traces. Combining those data with Figure 2b,d, we suppose that 180-s processing leads to (1) Cu/PS1 nanocomposite formation with a prevalent location of copper deposit as a film in the near surface region of the porous layer and (2) PS2 conversion into a porous copper layer which is partially covered with separated Cu particles of 160-nm average diameter. So, the Cu deposit structure greatly depends on the type of PS template. The porosity and thickness of PS are managed by anodic current density and time of anodization, respectively [29]. The distance between pore centers is a constant parameter [29]. An increase of current density leads to the increase of pore channel diameter. As a result, the porosity of PS increases simultaneously with the thinning of the pore walls. It is very likely that the complete displacement of PS2 is caused by a better reagent exchange in conditions of wider pore channels and smaller elements of Si skeleton in PS.

Formation of copper particles of the nanoscale range on the outer PS surface requires the use of PS of only 1-μm thickness. A thin porous layer allows minimizing the amount of reagents and deposition time needed for the growth of NPs [22]. However, that limited the thickness of the converted porous copper film just to 1 μm. In trying to study the properties of such porous copper, we separated it from the Si substrate, but the metallic film had too weak mechanical strength and, in free form, represented pieces of about 25-mm^2^ area. Thus, to further work with the free porous copper, the increase of its thickness was highly required.

**Figure 4:**
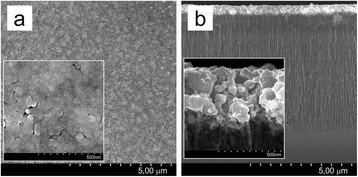
**Top (a) and cross-sectional (b) SEM views of quasi-continuous Cu film.** Cu film was grown on PS3 of 7-μm thickness and 80% to 85% porosity by displacement deposition for 1020 s.

**Figure 5:**
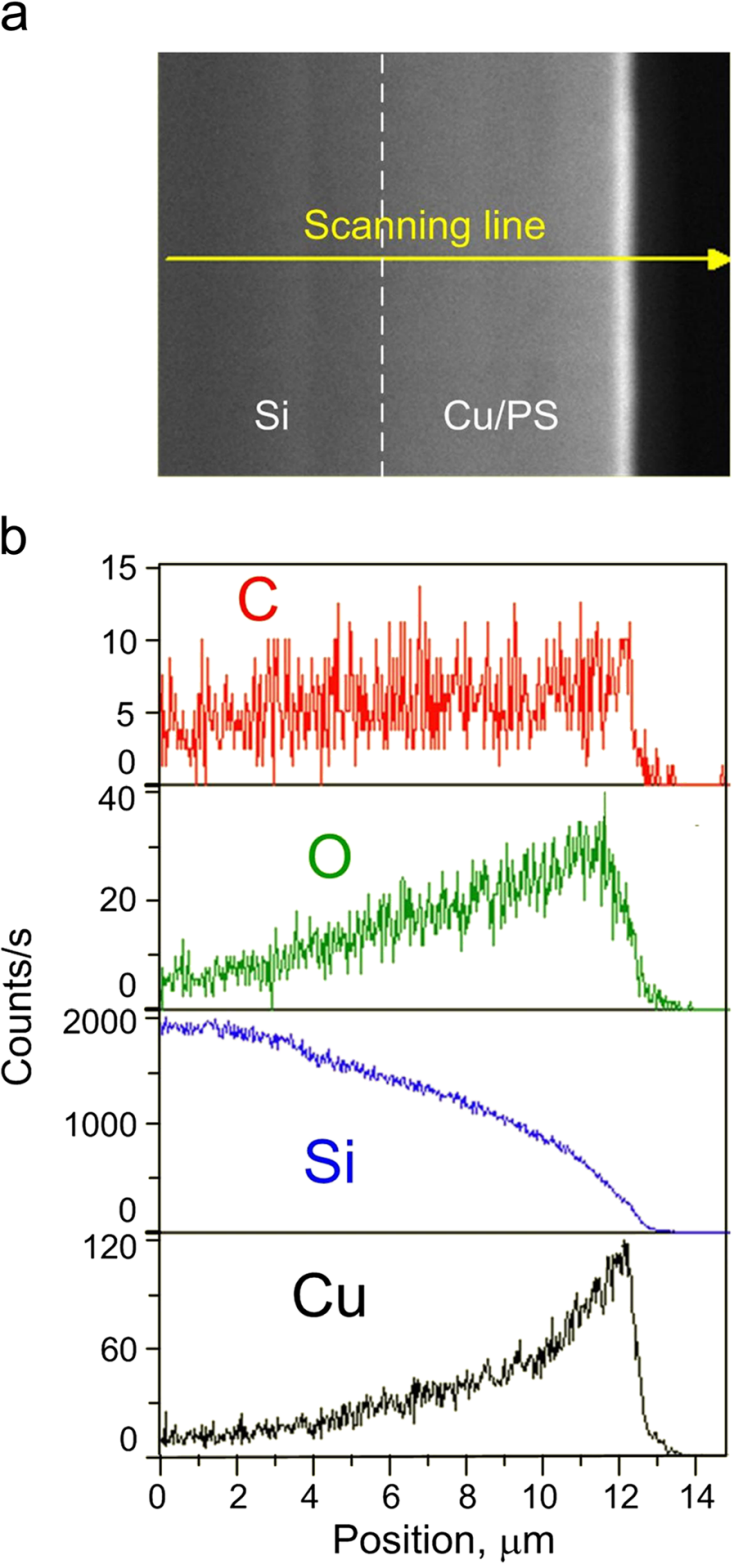
**EDX scan of the cross section of Cu/PS3.** (**a**) SEM cross-sectional view, (**b**) EDX data obtained by scanning along the yellow line.

Supposing the formation of a thicker layer of porous copper, we used PS3 (see Table 1) in connection with prolonged copper displacement deposition. The porosity of PS3 was the same as that of PS2, but the pores deepened up to 7 μm with increased anodization time. Visual monitoring of Cu deposition process showed the formation of copper deposit on the outer surface of PS3. Starting from 900 to 1020 s, we did not observe the evolution of hydrogen bubbles, so the time of PS3 immersion was limited to 1020 s. Figure 4 shows SEM images in top (a) and cross-sectional (b) views of PS3 immersed into the basic solution for copper deposition for 1020 s. The same sample was analyzed by XRD in [25] which revealed polycrystalline copper presence in its composition as well as small amounts of Cu_2_O. The top of the porous layer is covered with a noncontinuous copper film which consists of coalesced particles. The correct evaluation of particle diameters is difficult because their boundaries are unclear, but some of them might be measured as 40 to 50 nm in diameter (Figure 4a). The thickness of the film does not exceed 450 to 500 nm (Figure 4b). The dissolution of PS3 took place as its thickness decreased from 7 to 5.8 to 6 μm. To find the depth of copper penetration into PS3, energy-dispersive X-ray spectroscopy (EDX) scanning was performed (Figure 5). Despite metal penetration to the bottom of pores, the content of copper in the porous material was too small in comparison with silicon and decreased from the entrance to the bottom of pores. The presence of oxygen is probably caused by the formation of Cu_2_O, SiO_2_, and O_y_-Si-H_x_ as well as the small amount of penetrated carbon from the air during sample drying. We suppose that the distribution of copper in PS3 is caused by poor and slow exchange of reagents in the depth of pores coupled with the rapid growth of the copper film at the top of the porous layer, i.e., unevenness of the displacement process rate along the pore length. Finally, the upper metallic film closes the entrances of pore channels and prevents further redox reactions in porous volume. We carried out an additional experiment connected with the increase of the porosity of PS, but it led to the destruction of the PS skeleton. At the same time, decreasing the PS thickness to 3 μm did not provide a significant increase of copper amount as the upper copper film still formed faster than PS converted.

**Figure 6:**
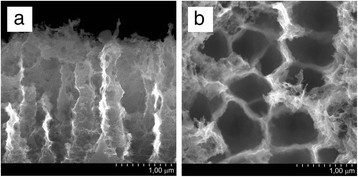
SEM cross-sectional (a) and top (b) views of PS4.

**Figure 7:**
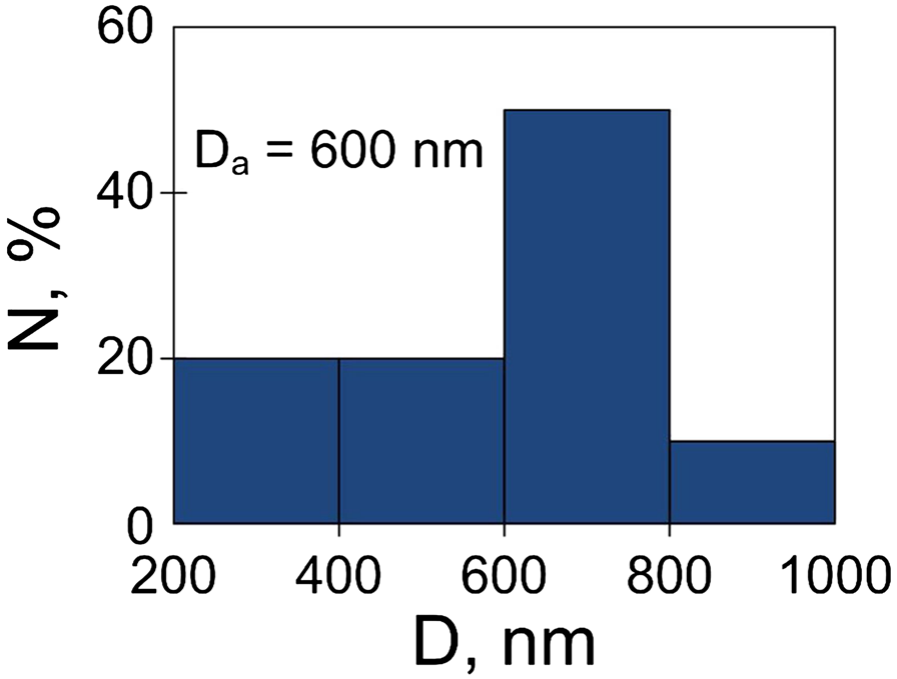
Pore size distribution histogram of PS4.

Therefore, to achieve a complete conversion of Si skeleton in copper, it is necessary to uniform the displacement rate along the pore channels. To meet the requirement, we increased the pore volume and improved the wettability of the surface of PS. The first change was connected with the use of macroporous silicon instead of mesoporous media. Macroporous Si has the same shape and order of pores as mesoporous materials, but the diameter of pores might be an order of magnitude greater [30]. Widening of pore channels provides better and more rapid penetration of reagents into the pore channels because diffusion limitation attenuates. Figure 6 shows SEM images in cross-sectional (a) and top (b) views of macroporous Si (PS4) prepared for conversion into copper which represents an ordered array of parallel pore channels. Pillar-like pore walls have bases wider than their tops and ragged surface. The thickness of porous layer was limited to 2.5 to 3 μm. This limitation was caused not only by the time of anodic treatment as in the case of mesoporous silicon. SEM analysis of several macroporous PS4 samples grown for different time periods was performed. PS thickness increase resulted in gradual thinning of the tops of Si pillars. In that way, porous layer reaching the thickness of 3 μm began to dissolve. The gravimetrically measured porosity of PS4 was 60% to 65%. Pore density (number of pores per square centimeters) was found from the evaluation of SEM images and varied in the range of 2 to 2.5· × 10^4^ cm^−2^. Figure 7 shows the distribution histogram of pore sizes of PS4. The common range of pore diameters is rather broad, but most part of the pores has a diameter of channel entrances varying in the range of 600 to 800 nm.

**Figure 8:**
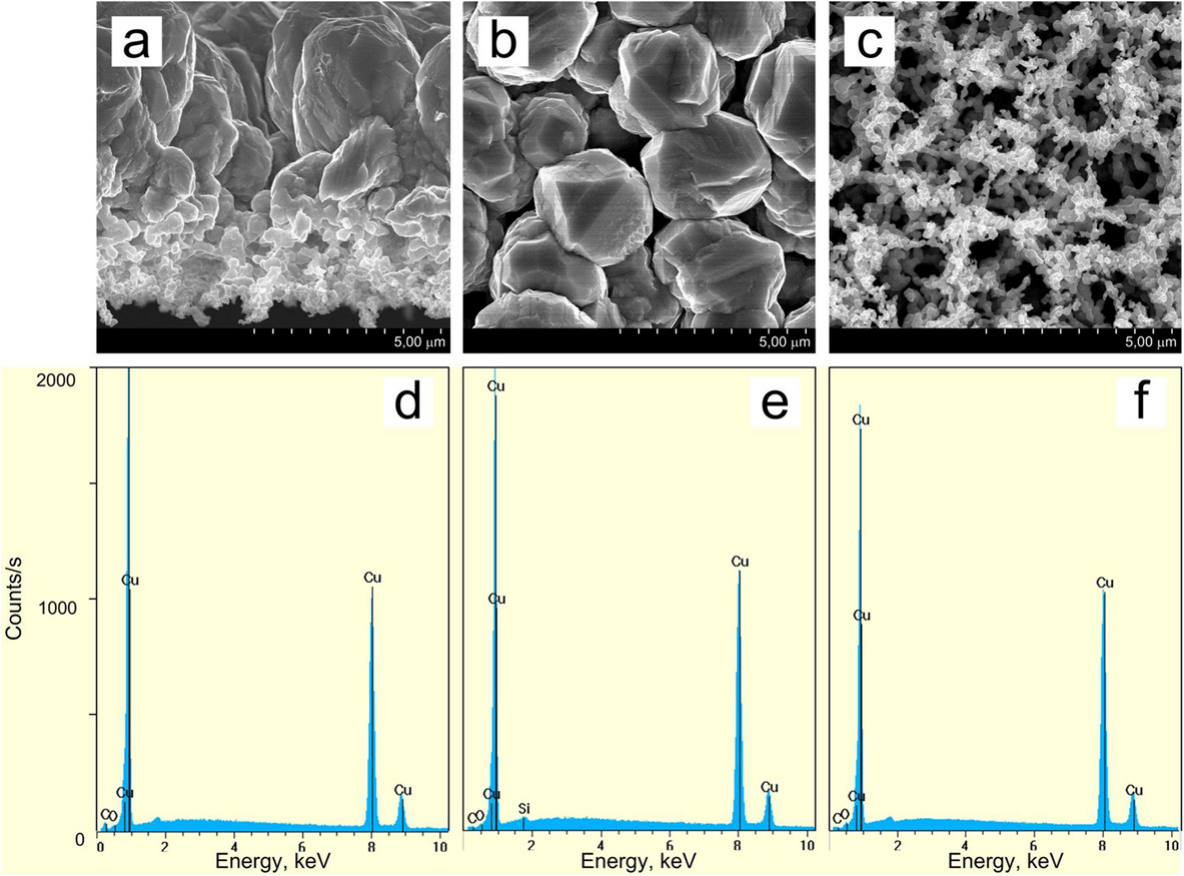
**SEM (a, b, c) and EDX point analyses (d, e, f) of the porous copper membrane.** The porous copper membrane was formed by displacement deposition of copper on PS4 for 7200 s from the solution of improved wettability; porous copper membrane was analyzed in cross section (**a**, **d**), top (**b**, **e**), and bottom (**c**, **f**).

**Figure 9:**
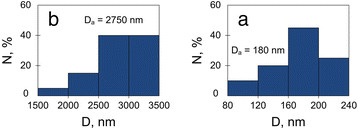
**Size distribution histograms of agglomerates and particles.** Histograms were calculated for (**a**) top and (**b**) bottom surfaces of the porous copper membrane.

Wettability improvement was achieved by adding isopropanol (C_3_H_7_OH) into the solution of copper salt. This alcohol significantly decreases the surface tension of water-based solutions [32], providing better contact between liquid and solid surfaces [33]. PS4 was left in the copper solution of improved wettability for 7200 s. Then, we observed the separation of the copper membrane from the Si substrate. The underlying Si had a surface without remains of porous layer. Figure 8 shows SEM images of the cross section (a), top side (b), and bottom side (c) of the separated membrane and related EDX point analysis which are considered in the next paragraph. The membrane represents a two-layered structure of 8-μm thickness. The top surface (Figure 8b) was the sample/solution interface, while the bottom (Figure 8c) was connected with the substrate. The top layer has a thickness of about 5 μm and represents a tightly packed array of parallel column-like agglomerates which are perpendicular to the substrate, i.e., columns grew along the pore direction of the original PS4. On the other hand, the bottom layer looks like a sponge of 3-μm thickness consisted of chains of small particles. Figure 9 presents the size distribution histograms of agglomerates and particles of the top and bottom surfaces of the membrane. Histograms were calculated from Figure 8b,c. The diameters of upper agglomerates are an order of magnitude greater than those of bottom particles. At the spongy layer, the particles of 160- to 200-nm diameters dominate. The prevalent diameter range of the upper agglomerates is 2500 to 3500 nm, but elements of two times less in diameters (to 1,500 nm) were found. The density of agglomerates was about 9 × 10^2^ cm^−2^, while the density of the bottom NPs was four orders of magnitude higher (9 to 16 × 10^8^ cm^−2^).

To reveal the elemental composition of the membrane, EDX analysis of the cross section, top side, and bottom side were carried out (Figure 8d,e,f). EDX scan of the cross section was attempted as well, but it was impossible to correctly focus the 1-μm electron beam on the non-flat surface of the agglomerates. To overcome doubts on the elemental composition, EDX analysis was performed in ten different points of the cross section, and each showed 97 to 99 at.% of Cu content. An example of point EDX in the cross section is presented in Figure 8e. Figure 6b,c confirms the copper nature of the obtained membrane. Overall, the membrane uniformly contains 95 to 99 at.% of copper with small amounts of oxygen and carbon. The maximum content of Si atoms was 0.1%, i.e., it might be declared that the obtained membrane represents the copper material. The gravimetrically determined porosity of the membrane was 60% to 65% in comparison with bulk copper.

Based on the results of SEM and EDX analyses, we propose the following phenomenological model of the formation of porous copper membrane. On the stage of full impregnation of PS with the solution, Cu NPs nucleate and grow on the surface of PS skeleton. As metal deposition was carried out simultaneously with dissolution of Si pillars, PS skeleton was converted into bottom spongy copper layer. The supposition might be proved by equality of the thickness of the original PS4 to that of the bottom copper layer (2.5 to 3 μm). In our opinion, new copper NPs grow and coalesce on the outer surface of the spongy copper layer. In that way, a layer of huge copper agglomerates is formed, whereas stresses on the Si/Cu membrane interface exceed over the interaction force between silicon and copper atoms when the copper membrane separates from the substrate as observed during the experiment. Detailed understanding of the porous copper membrane formation requires more careful in-depth study which is under the scope of the future paper.

**Figure 10:**
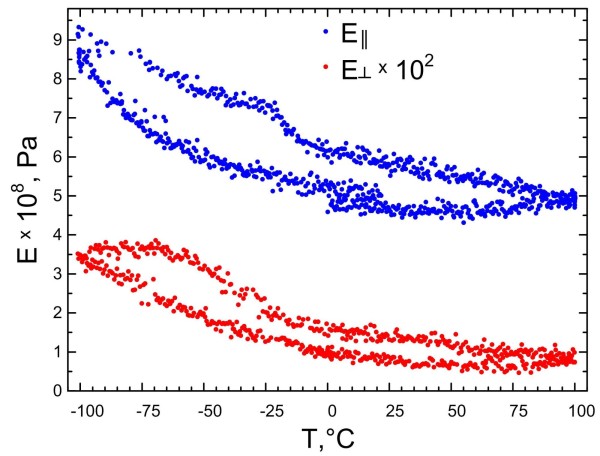
**Temperature variation of the Young modulus.** It was measured parallel to the pores or perpendicularly.

The temperature variation of the Young modulus *E*_*||*_ measured for the porous copper membrane during the flexural vibration and that of *E*_*⊥*_ measured during the extensional vibration are reported in Figure 10. In both directions, *E* increases at low temperatures, as usual in most solid samples. The measured values of the Young modulus (both *E*_*||*_ and *E*_*⊥*_) are much smaller than the value of *E* (110 to 128 GPa) for bulk polycrystalline copper, due to the high porosity and to the quasi-bidimensional feature of the membrane. It can also be noticed that the values of the Young modulus along the two directions differ by a factor of 300 at low temperature and 500 at high temperature, indicating a strong anisotropy of the sample, which is stiffer in the direction parallel to the pores, that is perpendicular to the plane of the membrane. Recently, a systematic experimental and theoretical investigation of the elastic constants and of the Young modulus of a block (approximately 10 × 10 × 10 mm^3^) of polycrystalline copper containing elongated pores was reported [34]. All crystallites had one crystallographic direction aligned along the [001] Cu axis and another two randomly oriented in the perpendicular plane. The pores were oriented along the [001] direction, and their diameters ranged between 15 and 380 μm. In such a system, the values of *E*_*||*_ and *E*_*⊥*_ strongly depend on the ratio of the axes of the ellipsoids associated to the pores. At low porosity (*p* < 20%), for pores having a high ellipticity, *E*_*⊥*_ *> E*_*||*_[34]. However, both values decrease with increasing porosity and virtually reach a null value for *p* = 100%. However, while *E*_*||*_ decreases linearly with *p*, *E*_*⊥*_ has a stronger dependence on porosity which leads to *E*_*||*_ > *E*_*⊥*_ for *p >* 20% [34] as in the case of the membrane investigated in the present paper. However, a quantitative comparison of the elastic modulus values between the membrane investigated in the present paper and the structure of [34] is not possible because the typical dimensions of the samples and of the pores differ by various orders of magnitude. Figure 10 also shows a clear hysteresis between cooling and heating in both vibration modes, which is reproduced upon subsequent cycling (results not shown) and could be possibly due to the absorption and desorption of gases on the porous structure.

**Figure 11:**
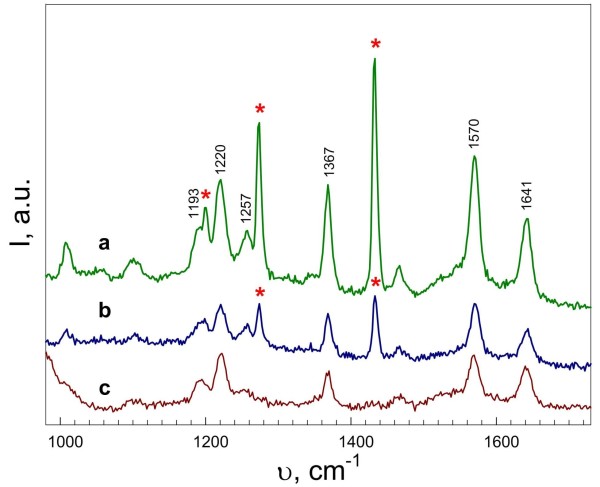
**SERS spectra.** (**a**) SERS spectrum of 10^−6^ M CuTMpyP4 adsorbed on the top side of porous copper membrane. (**b**, **c**) Raman spectra of 10^−6^ M CuTMpyP4 deposited from water and solution on bottom side of Cu membrane and porous silicon substrate, respectively. Laser plasma bands are marked by asterisks. Excitation wavelength was 441.6 nm.

The measurements of Raman spectra with water-soluble CuTMpyP4 as an analyte were performed for both sides of the porous copper membrane. Since water did not wet the surface of the porous Cu film (the solution formed a ball-shaped drop on the surface), the CuTMpyP4 precipitated from an aqueous-alcohol solution in a 1:1 ratio by volume. Figure 11 shows Raman spectra of CuTMpyP4 deposited from a 10^−6^ M solution on the top (a) and bottom (b) sides of the porous copper membrane and on PS without copper coating for comparison (c). It can be seen that distinct vibrational bands are observed in all spectra which correspond to CuTMpyP4 features reported in the literature [35,36]. The Raman intensity from the top side was three times of magnitude higher in comparison with that from the bottom side and from the porous surface of the PS sample. This observation reveals that the copper nanostructured surface fabricated on the top of PS by Cu displacement deposition exhibits some degree of SERS activity. In contrast, Cu porous layer from the bottom does not demonstrate enhancement of Raman signal. Comparing the 441.6-nm excited SERS spectrum of CuTMPyP4 (Figure 11a) with ordinary Raman spectrum in solid (Figure 11c), the close coincidence of the maxima wavenumbers and relative intensities of the bands can be observed. It means that no preferential orientation (*geometry of binding*) exists for CuTMPyP4 molecules adsorbed on the Cu membrane. The structure of the top of copper film was similar to the Si nanopillar array covered with copper that demonstrated the SERS activity in the paper [20]. So, the enhancement was likely to be caused by two reasons: (1) plasmon concentration on the tips of copper pillars and (2) the ‘hot spots’ in the copper pillar connections.

## Conclusions

Cu nanoparticles, quasi-continuous copper films, and free porous copper membranes were fabricated by displacement deposition of copper on PS templates from the aqueous solution of copper sulphate with HF and C_3_H_7_OH additions. It was found that the PS porosity and morphology as well as the time of deposition define the structural type of the Cu deposit.

The layers of mesoporous silicon of 1-μm thickness and 80% to 85% porosity represent a template for the fabrication of separated copper NPs of 20- to 280-nm diameter. Managing the Cu NP sizes is provided by time variation of PS immersion in the copper salt solution.

Copper displacement deposition on mesoporous silicon of 1- to 7-μm thickness and 50% to 85% porosity for more than 180 s allows formation of quasi-continuous copper films up to 500-nm thickness.

Macroporous silicon of 3-μm thickness and 60% to 65% porosity immersed into copper salt solution of improved wettability (with isopropanol additive) for 7200 s completely converts into porous copper membrane.

Young modulus of porous copper membrane depends on the porosity and has anisotropic nature in perpendicular and parallel directions. The measurements are useful for the further development of flexible and elastic materials for electroporation in biomedicine [20].

The demonstration of the modest SERS activity of the porous copper opens new prospects of Cu-based substrates for traces of substance detection. That might decrease the costs of SERS analysis in comparison with traditionally used substrates based on gold and silver.

## Abbreviations

EDX: Energy-dispersive X-ray spectroscopy; MEMS: Microelectromechanical systems; NPs: Nanoparticles; PS: Porous silicon; SEM: Scanning electron microscope; SERS: Surface enhanced Raman scattering; XRD: X-ray diffraction.

## Competing interests

The authors declare that they have no competing interests.

## Authors’ contributions

HB carried out the fabrication of Cu/PS samples, gravimetric measurements, and analysis of SEM images and XRD patterns, and designed and drafted the manuscript. SR and AS participated in the PS formation and studied the electrical characteristics of the experimental samples. AP and ST carried out the SERS measurements and spectra analysis. PN and MB carried out the copper porous membrane fabrication and Young modulus measurements. VB initiated, planned, and controlled the research process. All authors read and approved the final manuscript.

## Authors’ information

HB is a research scientist and is going to defend her Ph.D. thesis this fall 2012. SR is a junior researcher and a second year Ph.D. student. Both of them work in the R&D laboratory ‘Materials and Structures of Nanoelectronics’ (Micro- and Nanoelectronics Department, BSUIR). The head of the mentioned laboratory is VB, Ph.D., who is an assistant professor and teaches the courses ‘Nanomaterials’ and ‘Microelectronic Technology’. Sc. Dr. Professor AS is the head of the R&D laboratory ‘Information Display and Processing Units’ (Micro- and Nanoelectronics Department, BSUIR). AP, Ph.D., is a research scientist. ST, Ph.D., is a leading researcher in the R&D laboratory ‘Photonics of Molecules’ (B.I. Stepanov Institute, NASB). PN is a research scientist and is in the last year of being a Ph.D. student. Assistant Professor MB teaches the course ‘Microelectromechanical Systems.’ The two last authors are fellows of the Department of Information Engineering, Electronics and Telecommunications at the University “Sapienza.”
